# Metachronous Penile Metastasis From Advanced Anorectal Carcinoma: A Case Report

**DOI:** 10.7759/cureus.111110

**Published:** 2026-06-18

**Authors:** Muhammad Nabeel Shafqat, Ali Hassan, Muhammad Shahzad

**Affiliations:** 1 Internal Medicine, Three Crosses Regional Hospital, Las Cruces, USA

**Keywords:** anorectal cancer, anorectal malignancy, metachronous penile metastasis, metastatic rectal cancer, penile cancer

## Abstract

Secondary penile involvement in anorectal carcinoma represents an uncommon manifestation of advanced metastatic disease, with few cases documented in the literature. Despite the rich vascular and lymphatic supply of penile tissue, penile metastases arise predominantly from genitourinary primaries; an anorectal origin is exceptionally rare and is generally associated with poor oncologic outcomes. We report the case of a middle-aged man with T4N2M1 anorectal squamous cell carcinoma (SCC) and synchronous hepatic metastases who developed a painful ulcerative lesion and a firm subcutaneous nodule along the lateral penile shaft during concurrent chemoradiotherapy. Doppler ultrasonography identified a 1.4 × 1.4 cm hyperechoic, hypervascular lesion. Pelvic magnetic resonance imaging demonstrated multiple hypoenhancing lesions involving the corpora cavernosa, corpus spongiosum, and penile crura. Histopathological examination confirmed metastatic SCC with immunophenotypic concordance with the anorectal primary tumor. A multidisciplinary board recommended radical penectomy with perineal urinary diversion; however, due to rapid systemic disease progression and declining performance status, the patient transitioned to best supportive care.

Penile metastasis from anorectal carcinoma typically presents as a metachronous event and is indicative of widespread systemic dissemination with a guarded prognosis. Diagnosis is established through histopathological confirmation, supported by cross-sectional imaging to define the anatomical extent of disease. Treatment is primarily palliative and requires individualized multidisciplinary assessment that integrates disease burden, functional status, and patient-centered goals of care. Awareness of this rare metastatic pattern among clinicians managing pelvic malignancies is essential to facilitate prompt diagnostic evaluation and appropriate goals-of-care discussions.

## Introduction

Despite its rich vascular and lymphatic supply, penile metastasis from anorectal cancer is rare. Since its first description in 1870, penile metastasis has remained an uncommon clinical entity, with approximately 500 cases reported in the literature. Most cases originate from prostate and bladder cancers, whereas metastases from the lower gastrointestinal tract, including anorectal and colorectal cancers, are uncommon and account for only about 80 reported cases [1-3].

Anorectal squamous cell carcinoma (SCC) is the predominant histological subtype of anal canal malignancy, accounting for approximately 85% of anal cancers, with an annual incidence of 1-2 cases per 100,000 persons [3,4]. Unlike rectal adenocarcinoma, which drains predominantly through the portal venous system, anorectal SCC has a distinct multidirectional lymphovascular drainage pattern involving the mesorectal, hypogastric, and inguinal nodal basins, a feature that may facilitate diverse patterns of metastatic dissemination [5]. At initial presentation, distant metastases are observed in only 5%-8% of patients with anal SCC. However, following definitive treatment, the risk of distant recurrence increases to 10%-20%, with the liver and lungs representing the most common metastatic sites [6].

The proposed mechanisms of metastatic spread to the penis were first systematically described by Paquin and Roland in 1956 and include retrograde venous dissemination, retrograde lymphatic spread, arterial seeding, direct extension, and iatrogenic implantation [7]. Among these, retrograde venous spread through the periprostatic and pudendal venous plexuses remains the most widely accepted pathway, with the corpora cavernosa being the most commonly involved penile compartment [8]. Despite the rich vascularity of penile tissue, metastatic implantation is thought to be limited by the relatively unfavorable microenvironment of the corpora cavernosa, a concept consistent with Paget's “seed-and-soil” hypothesis, and typically occurs in the setting of advanced, widely disseminated pelvic disease [9].

Although rare, secondary penile malignancy is often fatal and generally signifies advanced disseminated disease. Penile metastasis from anorectal carcinoma is associated with a mean overall survival of approximately seven months [3,4]. However, recent advances in systemic therapy, including improved chemotherapeutic regimens and the emergence of targeted therapies, have contributed to better progression-free survival and improved management of treatment-related adverse effects in selected patients [10].

We present a case of metachronous penile metastasis arising from advanced anorectal SCC and provide a critical discussion of the available therapeutic strategies.

## Case presentation

We report the case of a man in his late 50s with a known diagnosis of metastatic anorectal SCC. He had locally advanced disease requiring a urostomy for the relief of malignant urinary obstruction and had multiple hepatic metastatic lesions. The cancer was staged as T4N2M1 and was being treated with concurrent chemotherapy and radiotherapy. At the time of diagnosis, physical examination revealed no penile lesions or nodules.

**Figure 1:**
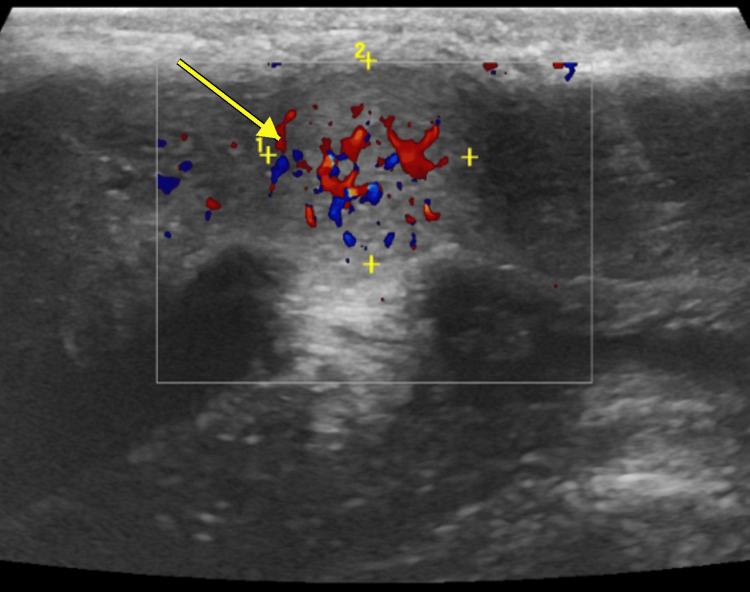
Penile shaft demonstrating subcutaneous edema with a focal 1.4 x 1.4 cm hyperechoic vascular structure in the left lateral region (arrow).

Approximately 11 months after the initiation of concurrent chemoradiotherapy, he developed a nodulo-ulcerative penile lesion with discharge. Physical examination revealed a penile ulcer and a hard, palpable subcutaneous nodule along the lateral aspect of the penile shaft. Given the aggressive nature of the anorectal carcinoma and the presence of hepatic metastases, the penile lesion was suspected to represent metastatic disease. Further evaluation with Doppler ultrasonography of the penile shaft demonstrated a focal 1.4 × 1.4 cm hyperechoic hypervascular lesion in the left lateral aspect of the shaft (Figure 1).

**Figure 2:**
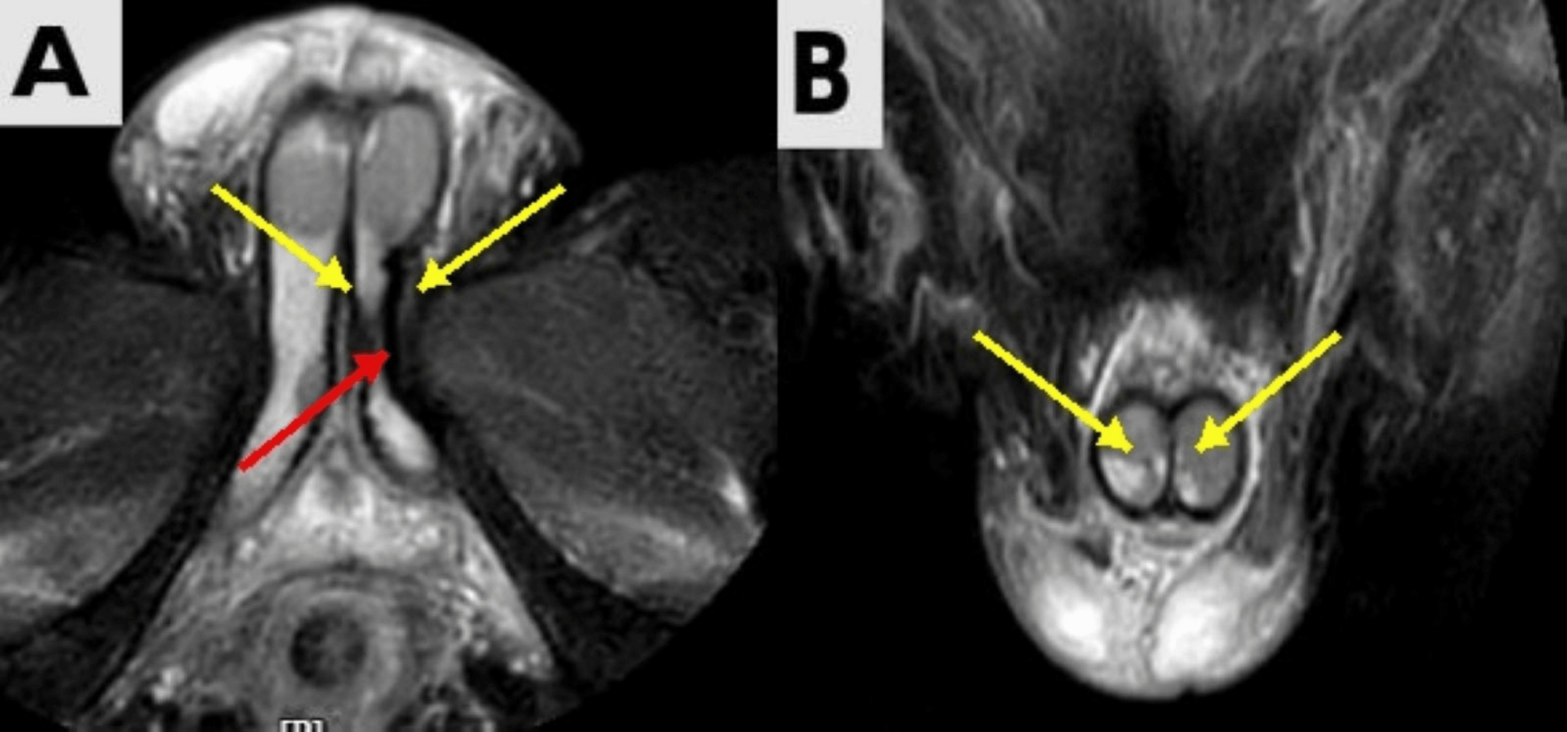
Multiple hypodense lesions involving the corpora cavernosum (yellow arrows) and corpus spongiosum (red arrow) on axial (A) and coronal (B) views.

Magnetic resonance imaging (MRI) of the pelvis was performed to further delineate the extent of metastatic involvement and characterize the penile lesions. MRI demonstrated multiple extensive T1- and T2-hypointense, hypoenhancing lesions involving the corpora cavernosa and corpus spongiosum, with extension into the penile crura (Figure 2). Biopsy of the penile lesions confirmed metastatic squamous cell carcinoma, consistent with the known anorectal primary. Based on the clinical, radiological, and histopathological findings, the patient was diagnosed with multiple penile metastases from anorectal carcinoma. The case was subsequently discussed at a multidisciplinary team meeting, where total penectomy with perineal urinary diversion was recommended. However, given the extensive disease burden, multiple hepatic metastases, and rapid clinical progression accompanied by declining performance status, the patient elected to pursue hospice care.

## Discussion

Anorectal SCC is a relatively rare malignancy, accounting for approximately 2% of all gastrointestinal cancers, with SCC representing the predominant histological subtype. Common metastatic sites include the liver, lungs, peritoneum, and lymph nodes. Penile metastases typically occur years after the initial diagnosis of anorectal or colorectal cancer. Despite the rich vascular and lymphatic supply of the penis and its anatomical proximity to pelvic organs, penile metastasis remains exceptionally rare. This paradox has been attributed to unfavorable conditions for tumor cell implantation within penile tissue. Metastatic spread most commonly occurs through retrograde venous or lymphatic dissemination from adjacent pelvic organs, particularly the bladder, prostate, and rectosigmoid colon, via established venous and lymphatic communications, as first systematically described by Paquin and Roland in 1956 [7]. Less frequently, arterial dissemination, direct extension, and iatrogenic implantation have been reported, particularly in cases arising from distant primary tumors. According to Paget's seed-and-soil hypothesis, the penile microenvironment is generally inhospitable to metastatic implantation; however, venous or lymphatic obstruction caused by extensive pelvic disease may facilitate tumor cell seeding. In the present case, retrograde venous dissemination facilitated by extensive pelvic tumor burden is the most likely mechanism underlying the development of penile metastases.

Approximately 500 cases of penile metastasis have been reported in the literature, most occurring metachronously and in association with advanced primary malignancies. Penile metastatic lesions are exceedingly rare as the initial manifestation of an underlying cancer and most commonly develop after the primary malignancy has already been diagnosed [11]. Clinical manifestations include penile nodules, malignant priapism, pain, and urinary symptoms. In patients with a known malignancy, these findings should raise suspicion for secondary penile involvement [12,13]. Bilateral involvement of the corpora cavernosa accounts for approximately 70% of penile metastases, whereas unilateral disease is uncommon and reported in only 15% of cases. Metastatic involvement of the corpus spongiosum and glans penis is even less frequent. The present case is particularly unusual because of unilateral metastatic involvement extending to the corpus cavernosum, corpus spongiosum, and penile crura [13].

Malignant penile lesions are evaluated using noninvasive imaging modalities such as Doppler ultrasonography, computed tomography, and MRI, which also assist in disease staging. However, histopathological examination remains the gold standard for differentiating primary penile malignancies from secondary metastatic lesions [14]. In the present case, Doppler ultrasonography and pelvic MRI raised suspicion for malignant involvement, and subsequent biopsy confirmed metastatic squamous cell carcinoma.

To date, only a few cases of penile metastasis originating from anal canal SCC have been reported in the literature, underscoring the exceptional rarity of this metastatic pattern. Pinheiro et al. described a 74-year-old man with previously treated T4N0M0 anal canal SCC who developed bilateral metastases of the corpora cavernosa two years after achieving a complete response to chemoradiotherapy [15]. Imaging excluded systemic disease, and the metastasis was considered isolated. Total penectomy was therefore performed with curative intent, and the patient remained disease-free for four years before eventual progression [15]. This contrasts markedly with the present case, in which penile involvement developed in the setting of synchronous hepatic metastases and rapidly progressive systemic disease, rendering radical surgical intervention inappropriate. This comparison highlights the importance of individualizing treatment according to the extent of systemic disease. Surgical resection may provide a meaningful survival benefit in patients with isolated metachronous metastasis, whereas a palliative approach is more appropriate in the presence of widespread dissemination.

Metastatic anorectal and colorectal carcinomas are associated with a poor prognosis. While median overall survival for metastatic colorectal adenocarcinoma ranges from 11 to 30 months, data specific to anorectal SCC with penile metastasis remain extremely limited. Survival estimates derived from rectal adenocarcinoma series, in which the mean overall survival is approximately seven months, may not be directly applicable to anorectal SCC because of differences in tumor biology and treatment responsiveness. Most reported cases of penile metastasis arise from rectal carcinomas, whereas metastases originating from colonic primaries are exceedingly rare, with only 10 cases reported since 1952 [15,16]. Novel systemic therapies, including immune checkpoint inhibitors targeting the PD-1 pathway, have demonstrated promising activity in metastatic anal SCC and may offer incremental survival benefits in selected patients.

## Conclusions

Penile metastasis from anorectal SCC is exceedingly rare and reflects advanced systemic disease with poor prognosis. New penile lesions in patients with known pelvic malignancies should prompt immediate evaluation for metastatic involvement. Early diagnosis allows timely multidisciplinary decision-making and appropriate alignment of care with patient goals.

## References

[1] Dekker E, Tanis PJ, Vleugels JLA, Kasi PM, Wallace MB (2019). Colorectal cancer. Lancet.

[2] Baidoun F, Elshiwy K, Elkeraie Y (2021). Colorectal cancer epidemiology: recent trends and impact on outcomes. Curr Drug Targets.

[3] De Rose AF, Vecco F, Gallo F (2023). Penile metastasis of colorectal cancer: case report and discussion on this rare clinical entity. Ann Case Report.

[4] Kuliavas J, Dulskas A, Drachneris J, Miseikyte-Kaubriene E, Samalavicius NE (2018). Penile metastasis from rectal carcinoma: case report and review of the literature. Visc Med.

[5] Biller LH, Schrag D (2021). Diagnosis and treatment of metastatic colorectal cancer: a review. JAMA.

[6] English KJ (2024). Anal carcinoma - exploring the epidemiology, risk factors, pathophysiology, diagnosis, and treatment. World J Exp Med.

[7] Paquin AJ, Roland SI (1956). Secondary carcinoma of the penis; a review of the literature and a report of nine new cases. Cancer.

[8] English K, Erpelding M, Kaldas S, Semoin S (2024). A case of a rare type of cancer: anal squamous cell carcinoma in a patient without significant risk factors. Qatar Med J.

[9] Fidler IJ (2003). The pathogenesis of cancer metastasis: the 'seed and soil' hypothesis revisited. Nat Rev Cancer.

[10] Gunderson LL, Winter KA, Ajani JA (2012). Long-term update of US GI intergroup RTOG 98-11 phase III trial for anal carcinoma: survival, relapse, and colostomy failure with concurrent chemoradiation involving fluorouracil/mitomycin versus fluorouracil/cisplatin. J Clin Oncol.

[11] Patel M, Kaldany A, Tanko F, Parrott A, Jang TL (2025). Penile metastasis as the presenting symptom of colorectal carcinoma: a rare case report. Case Rep Urol.

[12] Cocci A, Hakenberg OW, Cai T (2018). Prognosis of men with penile metastasis and malignant priapism: a systematic review. Oncotarget.

[13] Mearini L, Colella R, Zucchi A, Nunzi E, Porrozzi C, Porena M (2012). A review of penile metastasis. Oncol Rev.

[14] Santos Gda C, de Alvarenga ML, Borlot VF, Moutinho MA, de Franco MF (2009). Penile metastasis of urothelial carcinoma diagnosed by fine-needle aspiration. Cytojournal.

[15] Pinheiro AM, Pereira F, Duarte S (2024). Penile metastasis from anal canal carcinoma: a case report. J Urol Surg.

[16] Yin GL, Zhu JB, Fu CL, Ding RL, Zhang JM, Lin Q (2022). Metachronous isolated penile metastasis from sigmoid colon adenocarcinoma: A case report. World J Clin Cases.

